# Charting the Future: The Role of AI in Transforming Nursing Documentation

**DOI:** 10.7759/cureus.57304

**Published:** 2024-03-31

**Authors:** Abdulqadir J Nashwan, Ahmad Abujaber, Sirwan K Ahmed

**Affiliations:** 1 Nursing Department, Hamad Medical Corporation, Doha, QAT; 2 College of Nursing, University of Raparin, Ranya, IRQ

**Keywords:** decision support systems, data privacy, ethical considerations, artificial intelligence, nursing documentation

## Abstract

This editorial delves into the integration of artificial intelligence (AI) into nursing documentation, emphasizing its potential to streamline workflows, reduce human error, and enhance patient care. AI technologies, notably natural language processing and decision support systems, present opportunities to automate tedious documentation tasks and enhance record accuracy. However, their adoption raises ethical considerations, such as privacy, bias, and accountability. Striking a balance between technological advancements and ethical imperatives is pivotal to harnessing the benefits of AI while safeguarding patient safety and upholding professional integrity in nursing practice. Advocating for ongoing evaluation, regulation, and education is crucial to ensure the responsible integration of AI into nursing documentation. This approach aims to improve patient outcomes and maintain the high standards of the nursing profession.

## Editorial

Nurses dedicate a considerable portion of their work hours to documentation, with an average of 19% to as much as 35% of their time, varying by shift [1]. Morning shifts see the highest documentation time at 50.4%, with afternoon and night shifts slightly lower at 40.7% and 37.9%, respectively [2], which may even exceed the time spent on direct patient care that ranges between ​27% and 37% [3].

Artificial intelligence (AI) stands to significantly improve nursing documentation, streamlining workflows, and enhancing patient care. First, AI can automate the tedious aspects of documentation, allowing nurses to spend more time on patient care. Using natural language processing, AI can transcribe voice notes, interpret patient information, and populate fields accordingly [4]. It can also provide decision support, suggesting diagnostic codes based on documented symptoms or recommending interventions. Moreover, AI systems can track and predict patient outcomes, providing more accurate and timely documentation.

In the AI-powered nursing documentation field, software solutions are revolutionizing clinical workflows and improving patient care. IBM Watson Health utilizes advanced algorithms to quickly transcribe clinical dialogue into text. This software seamlessly integrates with electronic health record (EHR) systems, streamlining note completion. Dragon Medical, developed by Nuance Communications, provides real-time suggestions and simplifies documentation tasks, ensuring adherence to Health Insurance Portability and Accountability (HIPAA) compliance standards. Suki.ai employs natural language processing to generate comprehensive patient care documentation without the need for manual note-taking. It offers customizable features and integrates seamlessly with EHR/electronic medical record (EMR) platforms. Health Catalyst and Health Fidelity utilize AI-driven analytics to support population health management and risk stratification. These tools allow nursing professionals to identify at-risk patients and implement targeted interventions. Similar to Lindy, Freed, DeepScribe, and DeepCura, these AI-powered solutions prioritize accuracy, efficiency, and security in nursing documentation, enhancing the quality of care delivery in academic settings.

One of the primary benefits of AI in nursing documentation is reducing human error. AI systems can identify inconsistencies or omissions in patient records, prompting nurses to verify and correct information. This can lead to a higher standard of record accuracy and patient safety. Furthermore, predictive analytics can anticipate patient complications, allowing preventative measures to be documented and acted upon proactively.

However, integrating AI into nursing documentation raises significant ethical issues. Privacy and data security are paramount concerns [5]. While integrating AI into nursing documentation could enhance accuracy and efficiency, there's a risk that AI may record inputs as done or completed that were, in fact, not carried out. Moreover, AI systems require vast amounts of data to learn and function effectively, which could increase the risk of data breaches or misuse of sensitive patient information. There must be stringent measures in place to ensure that patient confidentiality is maintained and that data are used responsibly.

Bias is another ethical concern [4]. AI systems are only as unbiased as the data they are trained on. If historical data contains biases, AI could perpetuate or even exacerbate these biases, leading to unequal care. Ensuring that AI is trained on diverse, representative datasets mitigates this risk.

The question of accountability is also pivotal. When AI assists or even directs certain aspects of nursing documentation, it must be clear who is responsible for the outcomes - whether the nurse, the healthcare institution, or the AI developers. Nurses should understand the capabilities and limitations of AI to ensure they do not over-rely on its guidance. Policy-makers are tasked with establishing and enforcing regulations that mitigate the over-reliance on AI, treating it as an aide rather than a substitute for clinical judgment. Concurrently, healthcare institutions bear the responsibility of enacting policies and safeguards to orchestrate the judicious utilization of AI's decision-making capabilities.

Moreover, using AI in nursing documentation must consider the impact on the nursing profession. There is a potential for job displacement or deskilling of nurses if AI is seen as a replacement rather than a tool to augment nursing practice. There must be a focus on education and training to ensure that nurses can work effectively alongside AI.

Finally, there is a need for ongoing evaluation and regulation. AI systems should be continually assessed for accuracy, effectiveness, and ethical implications, with adjustments made as necessary.

In conclusion, while AI offers promising advancements in nursing documentation, its implementation must be approached with caution and responsibility. Ethical considerations, such as privacy, bias, accountability, professional impact, and regulation, are paramount. Balancing the technological potential of AI with these ethical imperatives will be critical to harnessing its benefits for improving patient care and nursing practice.
